# Spectrum and Impact of Mitochondrial DNA Mutations in Ovarian Cancer

**DOI:** 10.3390/ijms262211180

**Published:** 2025-11-19

**Authors:** Samantha Su Ping Low, Laura Greaves, Ryan Silk, Colin A. Semple, Charlie Gourley

**Affiliations:** 1 Nicola Murray Centre for Ovarian Cancer Research, Cancer Research UK Scotland Centre, Institute of Genetics and Cancer, University of Edinburgh, Edinburgh EH4 2XU, UK; 2 Mitochondria Research Group, Biosciences Institute, Faculty of Medical Sciences, Newcastle University, Newcastle upon Tyne NE2 4HH, UK; 3 MRC Human Genetics Unit, Institute of Genetics and Cancer, University of Edinburgh, Edinburgh EH4 2XU, UK

**Keywords:** mitochondrial DNA, somatic mutations, ovarian cancer, gene editing, heteroplasmy

## Abstract

Mitochondrial DNA (mtDNA) mutations are prevalent across cancer genomes, and growing evidence implicates their multifaceted role in energy metabolism with tumorigenesis. Ovarian cancer, in particular, demonstrates high mtDNA copy numbers and increased incidences of truncating and missense mtDNA mutations, with heteroplasmy levels predictive of prognosis. This review provides a comprehensive description of published mtDNA sequencing data in ovarian cancer, the majority being high-grade serous samples, encompassing both coding and non-coding regions. MtDNA mutations within non-coding regions, such as the D-loop control region, can affect mtDNA replication and transcription, hence affecting overall mtDNA copy numbers, while mtDNA mutations within coding regions can directly impact respiratory complex function and downstream metabolic pathways. MtDNA mutations may serve as clinically valuable diagnostic biomarkers for ovarian cancer and predictors for chemoresistance. We also explore ongoing efforts to deepen our understanding of mitochondrial oncogenetics through the creation of novel cancer models enabled by mitochondrial gene editing techniques. Developing robust human ovarian cancer cell models will be critical to elucidate mechanistic and phenotypic consequences of mtDNA mutations, assess drug response and resistance and identify new therapeutic targets to advance precision oncology in this emerging field.

## 1. Introduction

Mitochondria were initially observed in the 1850s and later named in 1898, drawing from the Greek words ‘mitos-’ meaning ‘thread’ and ‘-chondros’ meaning ‘granule’, inspired by their microscopic appearance [1]. These unique intracellular organelles are maternally inherited and possess their own genome, distinct to nuclear DNA (nDNA); they encode 13 proteins that are essential components of the subunits of the oxidative phosphorylation (OXPHOS) machinery [2]. The remaining mitochondrial proteins are encoded by nDNA and imported from the cytoplasm. Unlike the paired chromosomes of the human nuclear genome, mitochondrial DNA (mtDNA) is present in multiple copies in each cell, with cellular copy numbers ranging between 100 and 10,000, and replicates independently of the cell cycle [3].

Mitochondria play key roles in energy production, generation of reactive oxygen species (ROS), metabolic signaling and apoptosis. These varied functions not only grant mitochondria exquisite sensitivity to cellular stressors but also the ability to adapt to changes in their environment. Tumors leverage this versatility to support cancer cell initiation, proliferation, survival and metastasis in the often harsh climates of hypoxia, acidosis, nutrient depletion and anti-cancer therapies [4]. MtDNA is more susceptible to DNA damage than nDNA, with a background mutational rate 10–17 times higher, as a result of inefficient DNA repair mechanisms, proximity to ROS production and the lack of protective histones [5,6]. Coupled with high coding gene density and multiple genomic copies per cell, mtDNA provides a larger functional mutational target than nDNA.

Somatic mtDNA mutations are frequently seen in the cancer genome, including ovarian cancer, with approximately 50% of all tumors harboring at least one somatic aberration, most of which are heteroplasmic and at levels < 60% [7]. The mutational signatures observed are similar across tumor types, with a majority of C:G > T:A substitutions, followed by T:A > C:G substitutions [7]. A large whole-genome sequencing (WGS) study of 1916 patients across 24 cancer types found an overrepresentation of mtDNA variants in tumor genomes as compared to normal tissue [8]. In high-grade serous ovarian cancer (HGSOC) cohorts, higher rates of truncating and missense mutations have been identified in mtDNA compared to nuclear genes (with the exception of TP53, which is almost ubiquitously mutated in HGSOC) [9]. These deleterious effects of mtDNA mutations predict poorer patient prognosis [9]. Studies in ovarian cancer cells have also shown altered OXPHOS, increased mitochondrial biogenesis, a process where cells increase their mitochondrial mass, and changes in mitochondrial morphology [10,11].

MtDNA mutations are clearly present in cancer genomes, and it is conceivable how aberrations to mitochondrial function can modulate tumorigenesis. A deeper understanding of the underlying biology will enrich proposed efforts to target mtDNA mutations therapeutically in oncology. This review will provide a summary of the functional impact of mtDNA mutations, their role in ovarian cancer oncogenesis and the clinical implications of mtDNA mutations on diagnosis, chemoresistance and future therapeutic strategies.

## 2. Mitochondrial DNA: Structure and Genetics

The human mitochondrial genome is a small, double-stranded and closed DNA molecule consisting of 16,569 base pairs encoding 37 genes: 13 protein-coding, 22 tRNAs and 2 rRNAs. The 13 hydrophobic proteins insert into the inner mitochondrial membrane and form core subunits of respiratory chain complexes I, III, IV and ATP synthase [2]. MtDNA is composed of a guanine-rich ‘heavy strand’ and a cytosine-rich ‘light strand’ due to a disproportionate distribution of nucleotides across both strands [2]. The compact nature of mtDNA, due to the economical organization of genes, close together and often overlapping each other, results in little genetic redundancy. Nevertheless, there exists an approximately 1 kb long non-coding region (NCR) of mtDNA which regulates transcription and translation [3]. The displacement loop (D-loop) nests within the NCR and is a triple-stranded section of DNA made up of the heavy strand, light strand and a segment of partially replicated DNA known as 7S DNA. Replication of mtDNA is initiated within the D-loop [12].

Heteroplasmy is a phenomenon unique to mtDNA in non-plant eukaryotes. Human cells generally contain fifty to hundreds of mitochondria, each containing 5 to 10 mtDNA copies, hence amounting to a relatively large and varied total copy number of mtDNA within and between cells. Typically, only a proportion of mtDNA copies within a single cell are affected when mutations arise, generating a heterogenous mix of mutant and wildtype DNA described as heteroplasmy [13]. Homoplasmy is only present if every single copy of mtDNA is genetically identical within a cell. The level of heteroplasmy fluctuates with cell division due to random segregation of mutant and wildtype mtDNA molecules to the daughter cells, a process known as vegetative segregation. Even in non-dividing cells, mtDNA undergoes constant turnover, and individual molecules are selected at random for replication via a process known as relaxed replication. Both mechanisms can occur in concert to influence the mutational level of a cell through random genetic drift [14].

In the case of large-scale mtDNA deletions, selective clonal expansion of mutant mtDNA has been proposed to occur via replicative advantage of smaller mtDNA molecules with deletion mutations, as compared to larger wildtype counterparts [15,16,17], as well as negative feedback mechanisms that encourage an increase in mtDNA replication to compensate for deletion mutations causing reduced protein production [17]. The mechanism of clonal expansion of mtDNA molecules with point mutations is still unknown, but it has been shown computationally to fit with a pattern of random genetic drift [18,19].

The rate of mtDNA mutation and random genetic drift is likely to be significantly higher in rapidly dividing cancer cells than in their normal counterparts, resulting in a broad spectrum of mtDNA mutations in tumors. This could provide an opportunity to select mtDNA variants which favor cancer cell growth and survival. Cancer cells may have an increased tolerance for mtDNA variants due to their predilection for metabolizing glucose and lactate for energy production through glycolysis, known as the Warburg effect, instead of via the mitochondria-dependent OXPHOS [8,20]. However, near-homoplasmic or protein-altering mtDNA mutations can confer a negative selection pressure in some tumor types [21].

Epigenetics in mitochondria, coined ‘mitoepigenetics’, comprising mtDNA methylation or hydroxylmethylation, mitochondrial nucleoid modifications, mtRNA modifications and mtDNA-derived or nDNA-derived non-coding RNA modulations during mtDNA-encoded gene translation, have also shown a role in cancer development [22]. For example, decreased methylation of the D-loop region is seen in colorectal cancer cells as compared to benign tissues [23]. There also appears to be an inverse relationship between methylation levels of the D-loop region and mtDNA copy number and cancer cell proliferation in colorectal cancer, osteosarcoma and glioblastoma [23,24,25]. However, conflicting studies have also suggested that low levels of mtDNA methylation in colorectal adenoma do not affect mitochondrial gene transcription [26]. Further studies with a more precise methodology are needed to identify methylation sites on mtDNA to further understand the significance of mitoepigenetics in downstream mtDNA gene expression and its impact on tumor growth.

## 3. Ovarian Cancer and MtDNA

Ovarian cancer is the third most common gynecological cancer, with an incidence of 6.6% worldwide [27]. It is also the deadliest gynecological cancer, with a 5-year survival rate of only 17% in advanced ovarian cancer patients. This high mortality is largely due to patients predominantly presenting at advanced stages. Hereditary genetic mutation syndromes, such as BRCA1 and BRCA2 mutations and Lynch syndrome, are known strong risk factors for ovarian cancer [28].

More than 90% of ovarian cancers arise from epithelial cells, of which there are five main histological subtypes; high-grade serous, low-grade serous, endometrioid, clear cell and mucinous carcinomas. These subtypes have distinct genetic profiles and underlying biology, resulting in markedly different treatment responses and overall patient outcomes [29]. Unlike other cancers, which primarily metastasize via the hematogenous route, ovarian cancer cells metastasize either via local invasion of neighboring organs or are exfoliated and transported through peritoneal fluid, superficially seeding the peritoneal cavity [30]. Widespread intraperitoneal disease is often associated with recurrent ascites and bowel obstruction, especially if cytoreductive surgery is not performed or systemic drug treatment is ineffective. A small study of HGSOC patients with 22 matched plasma-to-tissue samples and 5 matched ascitic fluid-to-tissue samples showed that tumor-derived mtDNA mutations were more easily identified in ascitic fluid than plasma samples. This could reflect the cancer’s preferred mode of invasion and suggests a possible higher value in testing ascitic fluid instead of blood for mtDNA mutations [31].

HGSOC, the most common epithelial subtype, is characterized by significant nuclear genomic instability on a background of frequent homologous recombination deficiency (HRD), whole-genome duplication (WGD) or both [9]. Targeted therapies that have been developed are focused solely on nDNA mutations and their downstream effects. A clearer understanding of mtDNA alterations within ovarian cancer may open new doors for targeted treatments.

### 3.1. Mitochondrial DNA Alterations in Ovarian Cancer

There are several published studies which have sequenced the mitochondrial genome, either in whole or in part, in ovarian cancer (Table 1).

#### 3.1.1. Non-Coding Regions 

A small study of ten matched ovarian carcinoma and control pairs identified five unique somatic mtDNA mutations within the NCR in each of the five samples, all of which were homoplasmic. Notably, two of these samples harbored homoplasmic mutations within the D-loop [32]. The D-loop, which lies in the NCR, is felt to be a mutational ‘hot-spot’ from an evolutionary perspective due to presence of three hypervariable regions: HV1 (nucleotides 16,024–16,383), HV2 (nucleotides 57–372) and HV3 (438–574) [42,43]. Analysis of matched tumor and non-tumor samples from 49 ovarian cancer patients revealed that somatic and germline mtDNA single-nucleotide variants (SNVs) predominantly occurred within hypervariable regions, and their presence was associated with poorer prognosis compared to mutations in coding regions (CRs) [44]. This result was also echoed in a separate large dataset of more than 350 ovarian cancer patients, with negative selection seen against tRNA loop areas [40]. Analysis of 395 sequenced tumor samples from 35 HGSOC patients showed a significantly higher mutational frequency in the mtDNA D-loop as opposed to the mtDNA CR [31]. A further study reported subtype-specific mtDNA mutations within the NCR that could allow distinction between endometrioid and serous ovarian tumors [35].

Accumulation of D-loop mutations may have an impact on mtDNA copy number and expression, as the D-loop forms part of the control region which governs mtDNA replication and transcription [44]. The presence of D-loop mutations in ovarian cancer samples is associated with significantly higher mtDNA copy numbers than samples without D-loop mutations [31]. Correspondingly, mtDNA copy number is significantly elevated in ovarian carcinomas (range: 444–16,772 copies) compared with normal ovarian tissue [34]. Furthermore, ovarian cancer exhibited the most abundant mtDNA copy number in a large WGS project, generated by the International Cancer Genome Consortium (ICGC) and The Cancer Genome Atlas (TCGA), analyzing 2658 cancers across 38 tumor types [7]. A preliminary study of 38 primary epithelial ovarian cancers and 4 borderline ovarian tumors showed that mtDNA copy number was unrelated to disease stage but significantly higher in borderline, Grade 1, and Grade 2 tumors compared with Grade 3 tumors. Interpretation of subtype-specific differences was limited by the use of an outdated Type I/Type II classification, which does not adequately reflect the distinct cellular origins, genetics, and molecular features of each histological subtype [34].

Mutations at the 12S and 16S rRNA gene have also been identified in ovarian cancer [32,45]. Interestingly, no ovarian tumors were found to harbor somatic mutations at a highly polymorphic homopolymeric C stretch (D310) located within the D-loop, albeit seen in most other tumor groups [45].

#### 3.1.2. Coding Regions 

In 2012, a study looking at paired tumor and non-tumor samples in 5 different cancers identified both inherited and somatic mtDNA mutations within CRs of 28 ovarian serous cystadenocarcinomas. Among the 236 inherited and 14 somatic CR variants, non-synonymous variants were markedly more frequent in somatic than inherited mutations (93% vs. 31%). Heteroplasmy levels of somatic CR mutations ranged from 33.5% to 95.1% [36].

**Figure 1:**
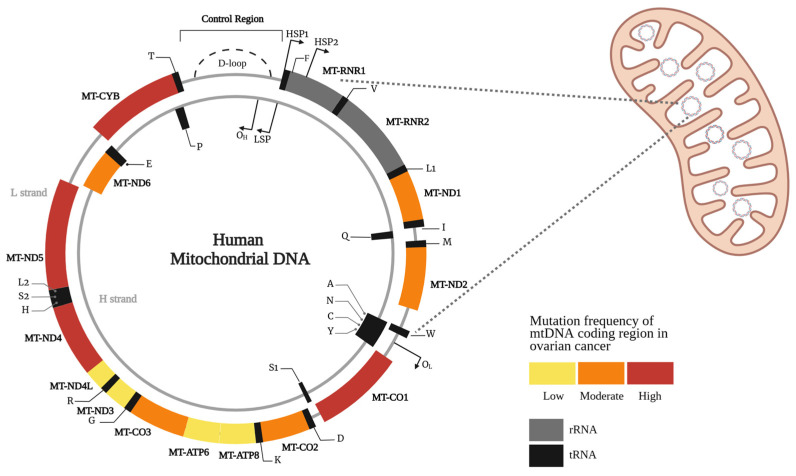
Distribution and relative frequency of somatic mitochondrial DNA mutations across coding regions in ovarian cancer. Color coding represents mutation frequency; red indicates high frequency, orange indicates moderate frequency and yellow indicates low frequency sites. This schematic is based on published data by Yuan et al. [7]. Created in BioRender. Churchman, M. (2025) https://BioRender.com/gcadyp4 (Accessed on 11 November 2025). Abbreviations: D-loop, displacement loop; HSP, Heavy Strand Promoter; LSP, Light Strand Promoter; mtDNA, mitochondrial DNA.

More recently, deeply sequenced HGSOC datasets have shown that predicted deleterious mitochondrial gene mutations affecting Complex I and IV function are most frequently implicated in HGSOC and occur at a higher rate than mutations in most nuclear tumor suppressor genes. The MT-ND5 gene, encoding NADH dehydrogenase 5—a subunit of Complex I—is the most commonly mutated mtDNA gene in HGSOC and across most other cancer types [7,9]. The aforementioned ICGC/TCGA WGS dataset also demonstrated that, among 113 ovarian cancer samples, mtDNA coding region mutations occurred most frequently in the MT-ND5, MT-ND4 and MT-CO1 genes [7] (Figure 1). Survival data has also shown that patients with HGSOC and predicted deleterious mtDNA somatic mutations have an inferior prognosis, with the extent of adverse outcomes directly correlating with increasing heteroplasmy levels, suggesting a gene–dosage effect [9]. However, exact tumor heteroplasmy levels were not reported in this study, limiting assessment of the upper heteroplasmy threshold to which this association remains true and of whether mutations nearing homoplasmy could instead impede cancer cell growth and potentially improve prognosis. Interestingly, synonymous mtDNA mutations and mutations within mitochondrial RNA genes were not associated with worse patient outcomes, emphasizing the damaging effects of compromised mitochondrial respiratory chain function.

MtDNA alterations occur more frequently within HGSOC tumors demonstrating WGD and less frequently in tumors demonstrating HRD [9]. Recent single-cell studies have revealed that elevated mtDNA copy numbers in HGSOC is strongly linked to WGD events in an attempt to maintain a balanced mtDNA-to-nDNA ratio [46]. The accumulation of mitochondrial DNA mutations and copy numbers hence appears to be differentially influenced by nuclear genomic instability, highlighting the complex interdependence between nuclear and mitochondrial genome dynamics.

A separate mtDNA sequencing study revealed an evolutionary pattern of greater mitochondrial genomic instability in epithelial ovarian cancer as compared to benign ovary tissue. This accumulation of somatic mtDNA mutations was characterized by a markedly lower proportion of pathogenic and likely pathogenic variants in genes encoding Complex V, compared with Complexes I, III, and IV [40]. The apparent negative selection against Complex V mutations underscores its critical role in ATP synthesis, contributing to cellular viability. Conversely, potential positive selection was observed in mutations within the MT-CYB gene of Complex III [40].

### 3.2. MtDNA Mutations in Primary vs. Metastatic Ovarian Tumors

Comparison of the mtDNA mutational pattern in paired primary and metastatic tumor samples of 35 HGSOC patients showed a significantly higher mutational density within the D-loop and higher mutation heteroplasmy levels in metastatic than primary tumors [31]. This suggests possible evolutionary selection of certain mtDNA mutations as tumors develop metastatic potential. No differences in mtDNA copy number or mutation density of CRs were found across paired samples [31]. Analysis of 17 bilateral ovarian tumor cases revealed 4 patients with distinct D-loop variants between paired tumors, suggesting independent clonal origins. Metastatic deposits, arising in these bilateral ovarian cancer cases, do however retain mtDNA variants identical to at least one of the primary tumors [33].

These mtDNA mutational signatures have been a useful tool to illustrate genetic divergence between primary and metastatic ovarian tumors. A linear metastasis model with low intratumoral genetic divergence and a parallel metastasis model with high intratumoral genetic divergence have been described in ovarian tumors. A small number of tumors demonstrated a mixed linear and parallel metastasis model. This translated to a poorer CA125 response in patients with parallel as compared to linear metastasis patterns and potential treatment resistance [31].

## 4. Clinical Utility of mtDNA Mutations in Ovarian Cancer

**Figure 2:**
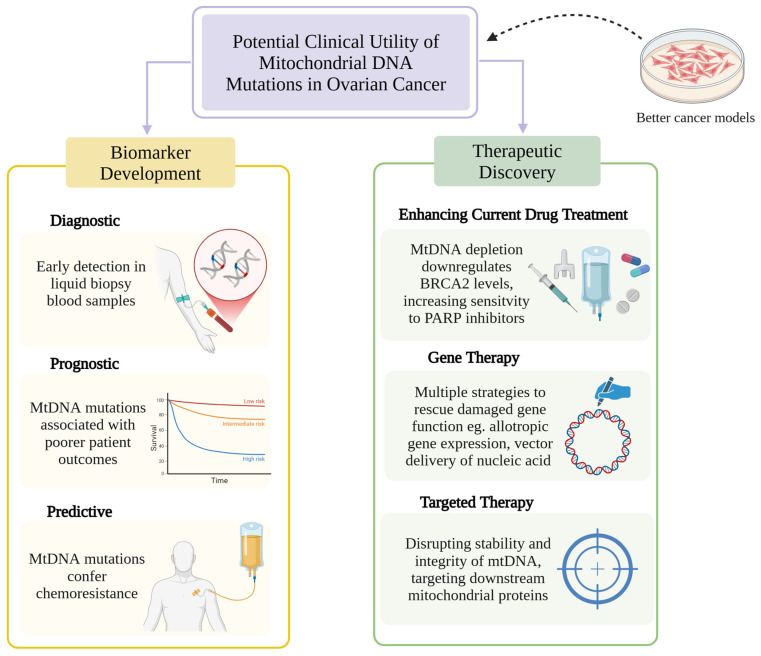
Potential clinical uses of mtDNA mutations in biomarker development and therapeutic discovery, aided by better human cancer cell models that can be created with novel mtDNA editing technology. Created in BioRender. Churchman, M. (2025) https://BioRender.com/e3i69fs (Accessed on 11 November 2025). Abbreviations: BRCA2, Breast Cancer Gene 2; mtDNA, mitochondrial DNA; PARP, Poly (ADP-ribose) Polymerase.

Understanding mitochondrial genomic variation and its significance in ovarian cancer has led to research exploring the translational application of mtDNA mutations in biomarker development and drug discovery (Figure 2).

### 4.1. MtDNA as a Cancer Biomarker

Biomarker development has been a burgeoning field of research in the new era of cancer therapeutics. Biomarkers can be used for screening, diagnosis, prediction of treatment response or resistance and prognosis across different malignancies. As efforts now move from the nuclear to the mitochondrial genome, greater insights into mtDNA alterations may lead to discovery of new cancer biomarkers [47,48,49]. A pilot observational retrospective study built a prediction model for HGSOC trained from mtDNA variations in 20 whole-exome-sequenced HGSOC samples and 14 controls, which was further validated with the TCGA dataset. This model identified that alterations in the cytochrome b gene, the only mitochondrial gene to encode a protein subunit of Complex III, increased the risk of HGSOC by over 30% [41]. The consequent expression of this gene, analyzed via RNA sequencing of paired HGSOC tumor and control specimens, was significantly decreased in this population. Continued efforts are needed to explore its potential role as a biomarker for early detection of HGSOC via liquid biopsy blood samples. While evaluation of somatic nDNA mutations such as mutant TP53 allelic fraction and reverse BRCA mutations in plasma cell-free DNA have demonstrated predictive and prognostic value in HGSOC, further research is needed to evaluate the utility of tumor-specific mtDNA mutations in plasma in this context [50]. Promisingly, mtDNA fragments have been detected in blood plasma and exosome samples in non-small-cell lung cancer, with the presence of exosome mtDNA closely associated with aggressive features of lung cancer [51].

Studies have also shown that the presence of single-nucleotide polymorphisms (SNPs) within the D-loop, identified from peripheral blood samples, are a risk factor and a predictive marker of age of onset, as well as a prognostic marker in epithelial ovarian cancer patients [37,38,39]. While not looking specifically for mtDNA mutations, increasing levels of detectable circulating cell-free mtDNA in peripheral blood are significantly associated with more aggressive epithelial ovarian cancer and poorer overall survival [52].

### 4.2. MtDNA Mutations and Chemoresistance

Platinum-based doublet chemotherapy has been the established first-line treatment for advanced ovarian cancer for nearly three decades [53]. Platinum agents, including cisplatin, carboplatin and oxaliplatin, induce intra- and inter-strand DNA crosslinking, which interferes with DNA replication and transcription, ultimately leading to cell death. This effect is seen in both nDNA and mtDNA, with some studies showing platinum agents having a greater affinity for damaging mtDNA over nDNA [54]. To escape chemotherapy-induced cytotoxic cell death, cancer cells must adapt to evade apoptosis. MtDNA mutations have been shown to confer chemoresistance through a variety of mechanisms, including metabolic remodeling, ROS generation, mito-nuclear crosstalk and mitochondrial translocation [55].

#### 4.2.1. Metabolic Remodeling and ROS Generation in Cancer Cells

A small sequencing study of 16 HGSOC patients found that patients harboring heteroplasmic pathogenic somatic mtDNA mutations exhibited higher rates of platinum resistance and cancer relapse [45]. These mutations, predicted to be deleterious or possibly deleterious by PolyPhen2 and SIFT, had heteroplasmy levels ranging between 8% and 73% and were all located within coding regions for Complex I and IV. Mitochondrial functional assays to evidence dysfunction confirmed a higher lactate-to-pyruvate ratio, indicating a shift from oxidative phosphorylation to glycolysis consistent with underlying mitochondrial dysfunction. On the contrary, platinum-sensitive patients had more synonymous somatic mtDNA mutations than their platinum-resistant counterparts [45].

Further evidence of metabolic remodeling was observed in the study of two human ovarian cancer cell lines—the 2008 cisplatin-sensitive cell line and C13 cisplatin-resistant cell line. MtDNA depletion in the 2008 cisplatin-sensitive cells led to platinum resistance as a result of mitochondrial dysfunction, while the cisplatin-resistant C13 cells showed decreased apoptosis, decreased mitochondrial membrane potential and lower basal oxygen consumption following platinum chemotherapy as compared to the 2008 cells [56]. Enhanced tumorigenesis with increased ROS generation and apoptotic resistance has also been associated with tumors derived from cybrids with heteroplasmic MT-ND5 and MT-ND6 mutations, both of which impact Complex I function [57]. Ovarian cancer cells possess a complex antioxidant defense system consisting of antioxidants such as NADPH, Nrf2, GST, GPxs, glutathione, peroxiredoxin and CD44v9 to protect themselves from mounting oxidative stress. The overexpression of these antioxidants in an attempt to neutralize extra ROS is often associated with an aggressive tumor biology, chemoresistance and poor survival outcomes in ovarian cancer patients [58].

#### 4.2.2. Mitochondrial–Nuclear Crosstalk

The mitochondria lie at a key intersection of nuclear and mitochondrial DNA and epigenetic control, facilitating bi-directional communication [59]. There is evidence to suggest that increased DNA methylation in gene promoter regions of post-chemotherapy ovarian cancer stem cells contributes to treatment resistance. Sensitivity to chemotherapy can be restored thereafter with the use of a DNA methyltransferase inhibitor, SGI-110 [60]. Epigenetic modifications are regulated in a dynamic manner by enzymes, environmental factors and cellular signaling pathways, with mtDNA mutations potentially contributing to this process. Cybrid models with a single mtDNA tRNALeu (UUR) m.3243G mutation showed distinct nuclear epigenetic changes at different levels of heteroplasmy. At high levels of heteroplasmy, a decrease in acetyl-CoA levels caused reduced histone H4 acetylation. At mid-levels of heteroplasmy, raised alpha-ketoglutarate levels was inversely correlated with histone H3 methylation [61]. This could suggest a mitochondrial–nuclear crosstalk basis for the development of chemoresistance through epigenetic modification of nDNA.

#### 4.2.3. Mitochondrial Translocation

Horizontal mitochondrial transfer is the phenomenon where there is cell-to-cell transfer of mitochondria and its corresponding mtDNA. Methods in which this can occur include the formation of tube-like structures called tunneling nanotubes (TNTs) or extracellular vesicles (EVs) from the donor cell to the recipient cell, facilitating mitochondria transfer, cell fusion, leading to the combination of organelles between two previously separate cells, and internalization of isolated mitochondria via endocytosis [62]. HMT allows cancer cells with defective or deleted mtDNA to overcome dysfunctional mitochondria-led respiration by transporting functional mitochondria from donor cells to cancer cells. This can restore normal respiratory function, reduce ROS levels, boost proliferation and cell migration and contribute to chemoresistance [55]. Platinum-resistant ovarian cancer cells displayed a greater tendency for TNT formation when cultured in hypoxic conditions as compared to platinum-sensitive ovarian cancer cells or benign epithelial cells, suggesting a role for mtDNA in development of chemoresistance under stress [63].

Mechanistically, senescent cell TNTs appear to be reliant on the mTOR signaling pathway and are partly mediated by downstream regulatory factor CDC42 [64]. In ovarian cancer cells, the EGFR/MAPK signaling pathway appears to be a major regulator of TNTs, with MEK and ERK inhibitors both reducing TNT formation and RSK inhibition showing reduced TNT numbers [65]. The use of Everolimus or Metformin, both mTOR inhibitors, has also shown preliminary evidence of suppression of TNT formation in chemoresistant ovarian cancer cells in vitro [63]. Other therapeutic strategies to disrupt HMT include EV inhibitors, for which heparin has shown an inhibitory role in EV uptake in ovarian cancer cells [66]. The process of cell fusion can be inhibited by targeting relevant signaling pathways such as NF-kB and VCAM-1/VLA-4 and proteins such as syncytin-1 and syncytin-2 [67,68,69,70].

#### 4.2.4. The Oncojanus Effect

To add more complexity, the concept of oncojanus, where certain genes can both promote and suppress tumor growth depending on context, has been seen with mtDNA mutations in ovarian cancer [71]. Below a certain level of heteroplasmy, mtDNA mutations which disrupt Complex I assembly are pro-tumorigenic; however, after exceeding a critical threshold level, severe dysfunction results in anti-tumorigenic effects. In a residual serous ovarian cancer case, a nearly homoplasmic novel missense mtDNA mutation in MT-ND4 was identified only in the post-chemotherapy surgical specimen which contained a previously undetected oncocytic component [72]. Considering the oncojanus effect, this novel MT-ND4 mutation could have initially bolstered chemoresistance within the tumor up until the threshold for Complex I disruption was reached, subsequently leading to the evolution of a slowly growing, benign oncocytic tumor [73].

### 4.3. Targeting mtDNA to Enhance Efficacy of Anti-Cancer Therapies

No mitochondrial-targeted anti-cancer therapies have yet been developed, but emerging evidence suggests that targeting mtDNA may enhance the efficacy of existing anti-cancer drugs. In prostate cancer cells, the presence of mtDNA depletion resulted in the downregulation of BRCA2 levels via activation of a calcium-mediated retrograde signaling pathway [74]. This confers enhanced sensitivity to Poly (ADP-ribose) polymerase (PARP) inhibitors, which, via synthetic lethality, exploit the already defective homologous recombination DNA repair process in BRCA2-depleted cells. This promotes accumulation of single- and double-stranded DNA breaks and leads to cell death. Further studies are required to determine whether this mechanism is applicable to ovarian cancer, particularly given the higher prevalence of mtDNA alterations observed in homologous recombination proficient tumors.

## 5. Developing Novel Cancer Models with mtDNA Mutations

Understanding the integral role mtDNA mutations play in tumorigenesis and chemoresistance has allowed ‘mitochondrial medicine’ to emerge as a novel opportunity to develop anti-cancer therapies against mitochondrial targets. There exists a broad spectrum of mitochondrial strategies, including therapies which directly target mtDNA itself to induce genetic damage and therapies that indirectly target mtDNA by influencing pathways involved in mtDNA stability and integrity, as well as therapies which target critical downstream mitochondrial proteins within metabolic pathways [73,75]. Despite multiple therapeutic strategies, only one gene therapy has successfully reached Phase III clinical trials to date—an AAV2 vector which promotes allotropic ND4 gene expression in patients with Leber’s hereditary optic neuropathy, a primary mitochondrial disease (PMD) associated with point mutations in MT-ND4 [76].

Although research into harnessing mtDNA mutations for therapeutic benefit in cancer remains in its infancy relative to PMDs, novel cancer models with mitochondrial mutations, which allow deeper biological insight and can guide future therapeutic strategies, are now being developed.

### 5.1. Mitochondrial Gene Editing

The impact of mtDNA on cancer biology has historically been studied using trans-mitochondrial cybrid models (donor cells providing mtDNA and recipient cells providing nuclear DNA) [77]. While insightful, this technology does not allow for specific mtDNA base editing to study the impact of cancer-causing mutations of interest. The advent of mitochondrial gene editing technology enables precise mtDNA engineering using DddA-derived cytosine base editors (DdCBEs) [78]. Derived from interbacterial cytidine deaminase toxins, DdCBEs induce C-G to T-A conversions in mtDNA. They consist of non-toxic split-DddA halves that activate only when combined with Transcriptor Activator-Like Effector (TALE) DNA-binding proteins at the target site. Isogenic mtDNA models can thus be generated for in vitro and in vivo analysis. Recent advances have led to the development of high-fidelity DdCBEs, which minimize off-target activities of conventional DdCBEs, caused by unwanted TALE-DNA interactions or spontaneous assembly of split DddA halves, thereby enhancing their precision and therapeutic promise [79].

### 5.2. Novel Cancer Models

While mtDNA manipulation has been successfully demonstrated in mice, zebrafish, human cells, human stem-cell-derived organoids and even chloroplasts, reports of mtDNA editing in cancer cells remain scarce [80,81,82,83,84,85]. An impactful study demonstrated the first application of DdCBE technology in cancer research by generating murine melanoma models with engineered truncating MT-ND5 mutations at heteroplasmy levels of 40%, 60% or 80% [86]. This study demonstrated heteroplasmy-dependent cellular redox imbalance favoring a Warburg-like shift towards aerobic glycolysis in mutant cells. Additionally, mtDNA mutants exhibited an altered tumor microenvironment with enhanced anti-tumor immune responses, with higher mutant heteroplasmy levels correlating with increased sensitivity to immune checkpoint inhibitors. These findings in mice models were mirrored in clinical data, with melanoma patients harboring mtDNA mutations at heteroplasmy > 50%, showing a 2.5-fold increase in response to Nivolumab, an anti-PD1 monoclonal antibody, compared with mtDNA wildtype patients [86]. To date, no published studies have reported the generation of isogenic human cancer cell lines, nor any work specifically in ovarian cancer.

## 6. Discussion

Ovarian cancers are characterized by frequent somatic mtDNA variants, particularly within CRs of mitochondrial respiratory chain complexes I and IV, as well as HV mutational ‘hotspot’ regions within the D-loop of NCRs. Further investigation of HV region variants is needed to elucidate the selective mutational bias toward these regions. Complementary RNA-sequencing analyses will be essential to validate their predicted impact on mitochondrial transcription and biogenesis, as accumulation of D-loop mutations correlates with elevated mtDNA copy numbers in ovarian cancer.

Definitive evidence for mtDNA mutations as direct driver events in tumorigenesis remains limited; however, such alterations likely provide cancer cells with a proliferative advantage over normal tissue. Metabolic reprogramming and elevated ROS production resulting from heteroplasmic mtDNA mutations may promote tumorigenesis and contribute to chemoresistance, underscoring mitochondrial dysfunction as a potential determinant of ovarian cancer progression. Moreover, mtDNA mutational patterns can reveal genetic divergence between primary and metastatic ovarian tumors, where greater divergence has been linked to poorer CA125 responses, supporting an oncogenic and possibly increased metastatic potential with accumulating mtDNA mutations. These mutations may also influence epigenetic regulation of nuclear genes, contributing to treatment resistance in post-chemotherapy ovarian cancer stem cells.

Despite growing interest in mtDNA variants in cancer, the literature on mtDNA variants specific to ovarian cancer remains limited, with most published studies involving modest cohorts. While genomic sequencing efforts have catalogued mtDNA variants in ovarian cancer, few have extended that to mechanistic or functional validation and its impact on oncogenesis. Research has largely centered on HGSOC, owing to its predominance among ovarian cancer cases. However, the distinct molecular and biological characteristics of each epithelial ovarian cancer histological subtype makes broad generalizations of mtDNA variants and its functional implications inappropriate.

Although mitochondrial gene therapy is advancing in PMDs—with one drug having reached clinical trials—no comparable mtDNA-directed therapies have been explored in cancer. Therapeutic progress is hindered by our limited and still-evolving understanding of the role mtDNA plays in cancer biology. While lessons may be drawn from drug discovery in PMDs, the complex interplay of numerous genomic alterations across both nuclear and mitochondrial DNA seen in cancer may make identification of a single therapeutic mitochondrial gene target challenging. Moreover, given the ubiquity and fundamental importance of mitochondria in nearly all human cells, off-target toxicities represent a major challenge for the development of mtDNA-targeted therapies. Encouraging progress has been made with the development of isogenic murine melanoma models carrying mtDNA mutations, but further work is required to extend this approach to other tumor types and human cancer cell lines.

## 7. Future Directions

Future research efforts should focus on the phenotypic characterization of mtDNA mutations for both NCRs and CRs in ovarian cancer with the use of novel cancer cell models developed with mitochondrial gene editing technology. This will allow for a deeper mechanistic understanding of the role of mtDNA variants in oncogenesis and help identify viable targets for new drug development. Additional research is warranted to explore the impact of mitochondrial–nuclear cross talk on cancer development, as well as the mitochondrial genetics underpinning tumor aggressiveness and treatment resistance. There is also an unmet need for mtDNA sequencing studies focusing on rarer ovarian cancer subtypes, such as low-grade serous and endometrioid ovarian cancer, in order to distinguish their mitochondrial mutational landscapes from that of HGSOC and examine their downstream impact.

Mitochondrial oncogenetics in ovarian cancer is still in its incipient stages. However, with precision oncology at the forefront of therapeutic discovery and patient management, we look to mtDNA mutations possibly emerging as the newest kid on the block, opening avenues for new targets and new treatments.

## Figures and Tables

**Table 1:** Studies that have sequenced mitochondrial DNA in ovarian cancer.

Author	Year	Histological Subtype (*n*)	Sequencing Method	Key Findings	Ref.
Liu et al.	2001	Primary ovarian cancer (*n* = 15) Primary ovarian cancer (*n* = 10)	PCR-based sequencing of D-loop PCR-based sequencing of complete mtDNA genome	Somatic mtDNA mutations in D-loop in 20% of samples. Somatic mtDNA mutations in NCR in 50% of samples, 20% in D-loop, all homoplasmic.	[32]
Trappen et al.	2006	Epithelial (*n* = 35, of which 17 are bilateral ovarian tumors)	PCR-based sequencing of D-loop	Somatic mtDNA mutations in D-loop in 26% of samples, all homoplasmic. In bilateral ovarian cancers, 24% showed different mtDNA variants between the paired tumors.	[33]
Wang et al.	2006	Serous (*n* = 19), endometrioid (*n* = 6), mucinous (*n* = 6), clear cell (*n* = 5), adenocarcinoma (*n* = 3), poorly differentiated (*n* = 3)	Real-time quantitative PCR	MtDNA copy numbers higher in ovarian tumor than normal ovary, not associated with age or tumor stage.	[34]
Aikhionbare et al.	2007	Serous (*n* = 42), endometrioid (*n* = 33), mucinous (*n* = 17)	PCR-based sequencing assessing D-Loop and 12S rRNA-tRNAphe, tRNAval, tRNAser, tRNAasp, tRNAlys, ATPase 6, ATPase 8, cytochrome oxidase I and II genes	Certain mtDNA mutations can distinguish between different histological subtypes of epithelial ovarian tumors: Stage 3 endometrioid: 12S rRNA gene (np A772T, 773delT, 780delT). Stage 4 serous: np 1657delC. Benign cystadenomas and borderline tumors: 8221delA.	[35]
Larman et al.	2012	Serous (*n* = 28)	WGS	236 germline variants identified (74 non-synonymous, 162 synonymous); 14 somatic variants identified (13 non-synonymous, 1 synonymous).	[36]
Kong et al.	2015	Serous (*n* = 27), endometrioid (*n* = 7), mucinous (*n* = 7), others (*n* = 19)	PCR-based sequencing of D-loop	SNPs (np 309, 324) in D-loop identified as independent predictors of ovarian cancer prognosis.	[37]
Kong et al.	2016	Serous (*n* = 43), endometrioid (*n* = 11), mucinous (*n* = 8), others (*n* = 27)	PCR-based sequencing of D-loop	SNPs (np 248, 524, 16,304) in D-loop are predictive markers for age-at-onset of ovarian cancer.	[38]
Liu et al.	2016	Epithelial (*n* = 93)	PCR-based sequencing	SNPs (np 73A.G, 207G/A, 523C/del) increased risk of epithelial ovarian cancer; SNPs (np 249A/del and 263A/G) decreased risk of epithelial ovarian cancer.	[39]
Xu et al.	2023	High-grade serous (*n* = 35)	Capture-based mtDNA sequencing	Metastatic ovarian tumors have a distinct mitochondrial genetic signature from primary ovarian tumors. Tumor-derived mtDNA mutations more likely detected in ascitic fluid than plasma.	[31]
Xie et al.	2024	Epithelial (*n* = 357)	Capture-based mtDNA sequencing	Negative selection against mutations in NCRs and Complex V genes. Potential positive selection for mutations in Complex III genes.	[40]
Ewing et al.	2025	High-grade serous (*n* = 324)	WGS, RNA sequencing	MtDNA mutations mostly in Complex I/IV genes and associated with prognosis; MT-ND5 most commonly mutated gene. Mutations seen particularly in WGD tumors.	[9]
Gonzalez Bosquet et al.	2025	High-grade serous (*n* = 20)	WES	MT-CYB variants predicted to increase risk of HGSOC by over 30%.	[41]

Abbreviations: D-loop, displacement loop; HGSOC, high-grade serous ovarian cancer; MtDNA, mitochondrial DNA, NCRs, non-coding regions; PCR, polymerase chain reaction; SNPs, single-nucleotide polymorphisms; WES, whole-exome sequencing; WGD, whole-genome duplication; WGS, whole-genome sequencing.

## Data Availability

No new data were created or analyzed in this study. Data sharing is not applicable to this article.

## Abbreviations

The following abbreviations are used in this manuscript:

AAV2	Adeno-associated Virus 2
BRCA	Breast Cancer Gene
CR	Coding Region
DdCBEs	DddA-derived Cytosine Base Editors
D-loop	Displacement Loop
ERK	Extracellular Signal-Regulated Kinase
EV	Extracellular Vesicle
GPxs	Glutathione Peroxidase
GST	Glutathione S-transferase
HGSOC	High-Grade Serous Ovarian Cancer
HMT	Horizontal Mitochondrial Translocation
HRD	Homologous Recombination Deficiency
HV	Hypervariable Region
ICGC	International Cancer Genome Consortium
MEK	Mitogen Activated Protein Kinase
mtDNA	Mitochondrial DNA
mTOR	Mammalian Target of Rapamycin
NADH	Nicotinamide Adenine Dinucleotide plus Hydrogen
NADPH	Nicotinamide Adenine Dinucleotide Phosphate plus Hydrogen
NCR	Non-Coding Region
NF-kB	Nuclear Factor Kappa-Light-Chain-Enhancer of Activated B Cells
Nrf2	Nuclear Factor Erythroid 2-Related Factor 2
nDNA	Nuclear DNA
OXPHOS	Oxidative Phosphorylation
PARP	Poly (ADP-ribose) Polymerase
PCR	Polymerase Chain Reaction
PMD	Primary Mitochondrial Disease
ROS	Reactive Oxygen Species
RSK	Ribosomal S6 Kinase
SIFT	Sorting Intolerant From Tolerant
SNP	Single Nucleotide Polymorphisms
SNV	Single Nucleotide Variant
TALE	Transcription Activator-like Effector
TCGA	The Cancer Genome Atlas
TNT	Tunnelling Nanotube
VCAM-1	Vascular Adhesion Molecule-1
VLA-4	Very Late Activation Antigen-4
WES	Whole-Exome Sequencing
WGD	Whole-Genome Duplication
WGS	Whole-Genome Sequencing
